# Reducing the potential for processing contaminant formation in cereal products

**DOI:** 10.1016/j.jcs.2013.11.002

**Published:** 2014-05

**Authors:** Tanya Y. Curtis, Jennifer Postles, Nigel G. Halford

**Affiliations:** Plant Biology and Crop Science Department, Rothamsted Research, Harpenden, Hertfordshire AL5 2JQ, United Kingdom

**Keywords:** Acrylamide, Furan, Processing contaminant, Asparagine, Reducing sugars, Maillard reaction, Free amino acids, Lipid oxidation, *Trans* fatty acids, Hydroxymethylfurfuryl

## Abstract

Processing contaminants may be defined as substances that are produced in a food when it is cooked or processed, are not present or are present at much lower concentrations in the raw, unprocessed food, and are undesirable either because they have an adverse effect on product quality or because they are potentially harmful. The presence of very low levels of processing contaminants in common foods is becoming an increasingly important issue for the food industry, as developments in analytical techniques and equipment bring foods under closer and closer scrutiny. This review considers the formation of lipid oxidation products, hydrogenation of polyunsaturated fatty acids to prevent lipid oxidation and the associated risk of *trans* fatty acid formation. The formation of acrylamide in the Maillard reaction is described, as well as the genetic and agronomic approaches being taken to reduce the acrylamide-forming potential of cereal grain. The multiple routes for the formation of furan and associated chemicals, including hydroxymethylfurfuryl, are also described. The evolving regulatory and public perception situations for these processing contaminants and their implications for the cereal supply chain are discussed, emphasising the need for cereal breeders to engage with the contaminants issue.

## Highlights

• Processing contaminants defined.
• *Trans* fat, acrylamide, furan and hydroxymethylfurfuryl formation described in detail.
• Genetic and agronomic approaches to reducing the potential for contaminant formation described.
• Evolving regulatory situation reviewed.
• Breeders urged to engage on the issue of processing contaminants.

## 1 Introduction

A food contaminant, based on dictionary definitions, may be described as a substance that makes a food impure. Foods are complex mixtures, of course, so it may be more useful to define a contaminant as something that would not normally be present in the food, has been introduced inadvertently from an external source or as a result of microbial, fungal or animal activity, or even just as something undesirable. Mycotoxins produced by fungal activity are an obvious example and are dealt with in another article in this volume. The issue becomes more complicated when food processing and cooking are involved, as these may bring about substantial changes in composition, including the production of substances that are not present in the raw food. Of course, these changes may be necessary to make the food edible and/or palatable, and when these substances are responsible for the colour, flavour and aroma of a food and have no negative effect on human health they are certainly not described as contaminants. Some of these compounds, however, do have the potential to have a negative effect on product quality, or even to cause harm, and therefore fall into the category of processing contaminant. We may therefore describe a processing contaminant as a substance that is produced in a food when it is cooked or processed, is not present or is present at much lower concentrations in the raw, unprocessed food, and is undesirable either because it has an adverse effect on product quality or because it is potentially harmful.

In this review, we describe four processing contaminants, *trans* fatty acids, acrylamide, furan and hydroxymethylfurfuryl, their implications for the cereal food supply chain, and the strategies that are being or could be developed to reduce their levels in cereal-derived products.

## 2 Thermal oxidation of lipids and the production of *trans* fatty acids

Wheat (*Triticum aestivum*), barley (*Hordeum vulgare*) and rye (*Secale cereale*) grain contains only approximately 1% oil and it is not worth extracting the oil for commercial use. Maize (*Zea mays*) and oat (*Avena sativa*) grain, however, contain around 5% and 7% oil, respectively. There is some commercial use of oat oil in cosmetics and skin moisturisers, but maize oil is a much more important commodity, with, for example, 1.3 billion litres of it being used in the USA in 2012, mostly for margarine and cooking oil (data from United States Department of Agriculture). Maize oil is made up of linoleic acid (54%), oleic acid (30%) and palmitic acid (12%), with stearic acid and α-linolenic acid making up the rest. Linoleic acid is an 18-carbon, omega-6 (18:2 n-6) polyunsaturated fatty acid, while oleic acid is an 18-carbon, omega-9 (18:1 n-9) monounsaturated fatty acid and palmitic acid is a 16-carbon saturated fatty acid (16:0). Polyunsaturated fatty acids are prone to oxidation during cooking and high-temperature processing, giving rise to lipid peroxides. These can form polymers that give a dark colouration and may be toxic. They can also breakdown to form various ketones, alcohols, hydrocarbons, acids and epoxides. These breakdown products cause a rancid, ‘off’ flavour and odour and can give rise to the formation of furans (Section 4).

Traditionally, food processors have avoided polyunsaturated fatty acid oxidation by chemical hydrogenation of the double bonds, converting the fatty acids to monounsaturates and saturates. Saturation of the fatty acids in plant oils also solidifies them, making them suitable for the production of margarines. The problem with the process is that some of the double bonds remain unsaturated but change from the *cis* form, which is present in the naturally-occurring molecule, to the *trans* form. In the *cis* form, the two hydrogen atoms attached to the carbon atoms involved in the double bond are on the same side of the double bond, while in the *trans* arrangement they are on opposite sides. The molecule is therefore identical to the naturally-occurring polyunsaturate in terms of length and number of double bonds but has a more linear shape. Nevertheless, it is regarded as so different from the *cis* form of the molecule that in the USA and Europe it cannot legally be designated as a polyunsaturate in food. This is partly because *trans* fatty acids arising from partial hydrogenation of plant oils are now regarded as being as harmful as saturated fatty acids in raising the level of low-density lipoprotein (LDL) cholesterol when consumed (reviewed by Brouwer et al., 2010) (it should be noted here that, in contrast to *trans* fatty acids produced in this way, vaccenic acid, a *trans* fatty acid found naturally in beef and dairy products, has been claimed to have health benefits (Bassett et al., 2010, Wang et al., 2010)).

The US government introduced legislation requiring information on the *trans* fat content of food products to be included in the Nutrition Facts panel of food labels in 2008 and, while there is no European Union legislation on *trans* fats, some Member States have acted independently. Denmark, for example, introduced legislation in 2003 limiting the *trans* fat content of fats and oils to 2%, with the limit applying to ingredients, not finished foods. The United Kingdom government, on the other hand, has not introduced legislation but there is a voluntary pledge from all major food manufacturers to remove *trans* fats from their products.

Soybean oil also contains a lot of linoleic acid and a genetically modified (GM) variety, Plenish, in which the activity of a gene encoding a *delta-12* desaturase enzyme that converts oleic acid to linoleic acid is reduced, has been produced by PBI, a subsidiary of DuPont. This variety accumulates oleic acid to approximately 80% of its total oil content, compared with 20% in non-GM varieties (Kinney, 1997), making the oil more stable during frying and cooking, less prone to oxidation and therefore less likely to form compounds that affect flavour (Mounts et al., 1994, Cahoon, 2003). Hydrogenation, with its risk of *trans* fatty acid formation, is not required. Monsanto also has a high oleic acid variety, Vistive, on the market; in this variety the high oleic acid trait was developed by mutagenesis, not GM, although the variety also carries a GM trait for tolerance of the herbicide, glyphosate. The technology has attracted some controversy because linoleic acid and its further desaturated derivative, α-linolenic acid, are important dietary fatty acids and consumers may not understand the significance of the changes that have been made. There are currently no reports of it being transferred to maize, despite the established market for biotech maize varieties in the USA and many other parts of the world. Nor are we aware of other non-GM approaches to reducing the risk of *trans* fatty acid formation.

## 3 Acrylamide

### 3.1 Chemistry of the Maillard reaction and formation of acrylamide

The Maillard reaction, which was named after the French chemist, Louis Camille Maillard, who first described it in 1912 (Maillard, 1912), comprises a series of non-enzymic reactions between sugars and amino groups, principally those of amino acids. It takes place only at high temperatures and occurs mainly in cooked foods prepared by frying, baking and roasting. The reaction requires a reducing sugar such as glucose, fructose or maltose, although sucrose can participate if it is first hydrolysed through enzymic, thermal or acid-catalysed reaction (De Vleeschouwer et al., 2009). The products of the reaction include melanoidin pigments, which are complex polymers that are responsible for the brown colour in fried, baked and roasted foods, including bread crust, biscuits, crackers, cakes, tortillas, fried tortilla chips and other snacks, and toasted grains. The reaction also provides complex mixtures of compounds that impart flavour and aroma, including pyrazines, pyrroles, furan (a contaminant described in Section 4), oxazoles, thiazoles and thiophenes (Mottram, 2007). The particular compounds formed give different cooked foods and brands their defining characteristics, and depend on the amino acid and sugar composition of the raw material and the processing conditions.

The reaction is initiated by the condensation of the carbonyl (C=O) group of a reducing sugar with the amino group of an amino acid or other amino compound, producing a Schiff base. The Schiff base cyclises, in the case of an aldose to produce an N-substituted aldosylamine, such as glucosylamine from glucose, then undergoes acid-catalysed rearrangement to an Amadori rearrangement product (or a related Heynes rearrangement product if the sugar is a ketose), followed by enolisation, deamination, dehydration and fragmentation to give rise to sugar dehydration and fragmentation products. These intermediates include furfurals, furanones and pyranones. They contain one or more carbonyl groups and are therefore highly reactive and can undergo further condensation reactions with amino groups and other species.

The Maillard reaction is very complex and more information can be found in reviews by Halford et al., 2011, Mottram, 2007 and Nursten (2005). While many of its products contribute to the flavour, colour and aroma of food, some are undesirable (Friedman, 2005). Indeed, as long ago as 1980, it was noted that the nutritional value of foods decreased after browning and that this must be caused by the generation of undesirable compounds (Yannai, 1980). It has since been discovered that one of the reactions of carbonyl compounds is Strecker degradation, whereby an amino acid is deaminated and decarboxylated to give an aldehyde and an α-aminoketone, and that a Strecker-type reaction involving asparagine results in the formation of acrylamide (Mottram et al., 2002, Stadler et al., 2002, Zyzak et al., 2003). This appears to be the major route for acrylamide formation, but others have been proposed, for example with 3-aminopropionamide as a possible transient intermediate (Granvogl and Schieberle, 2006) or through pyrolysis of gluten (Claus et al., 2006).

### 3.2 Risk posed by dietary acrylamide and the response of regulatory authorities

**Fig. 1:**
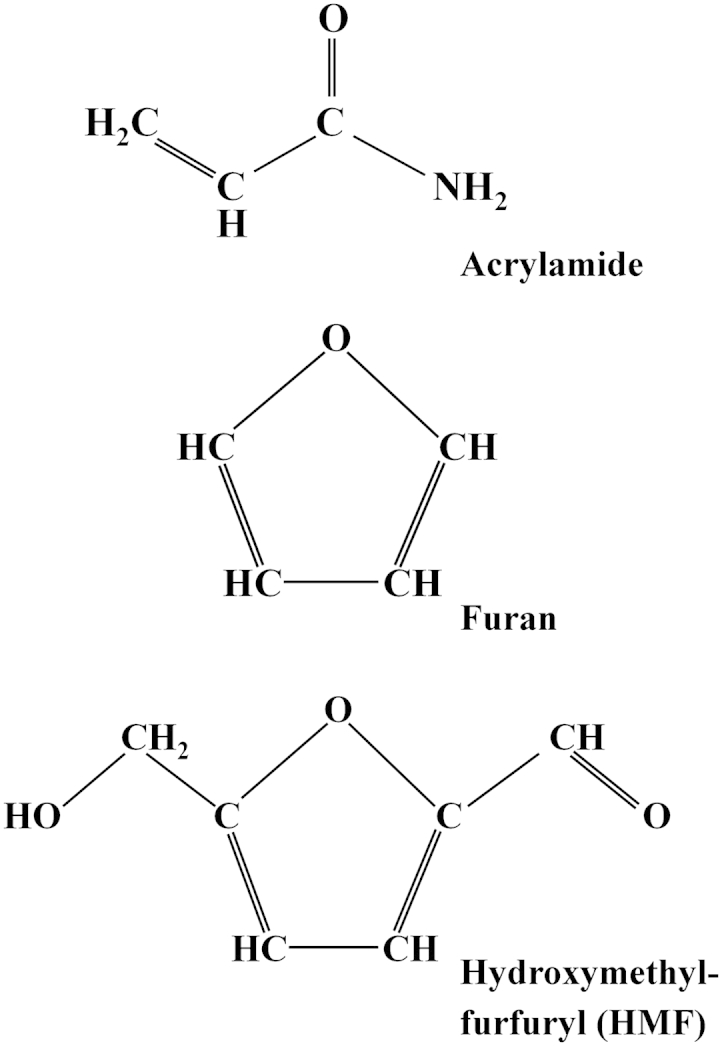
Diagrams representing the structures of acrylamide, furan and hydroxymethylfurfuryl.

Acrylamide (prop-2-enamide) (C_3_H_5_NO) (Fig. 1) is a familiar industrial chemical that is used in its polymerised form in wastewater treatment, gel electrophoresis, papermaking and fabrics manufacture. It has been shown to be carcinogenic and to have neurological and reproductive effects at high doses in rodent toxicology studies (Friedman, 2003, Taeymans et al., 2004). As a result, it has been classified as a Group 2A, ‘probably carcinogenic to humans’, chemical by the International Agency for Research on Cancer (International Agency for Research on Cancer, 1994). Both acrylamide and its primary epoxide metabolite, glycidamide, form adducts with haemoglobin and these can be used as a measure of exposure.

Acrylamide's use as an industrial chemical means that a tolerance level of 1 μg L^−1^ (part per billion) has been set for its presence in water, a concentration that is at the limit of detectability. It came as quite a shock for the food industry, therefore, when significantly higher levels were found in common foods. This happened in 2002 when a Swedish team that was investigating acrylamide exposure resulting from an industrial incident found evidence of exposure in its control group (Tareke et al., 2002). The cause was found to be dietary, with acrylamide being discovered in many common, plant-derived foods, including coffee and potato- and cereal-based products such as crisps, French fries, bread, cakes, biscuits, breakfast cereals, crisp-breads, maize snacks and more.

The food industry reacted quickly to the discovery and in the ensuing eleven years has devised many strategies for reducing acrylamide formation by modifying food processing (compiled in a ‘Toolbox’ produced by Food Drink Europe (2011)). They include modification of time/temperature conditions during processing, lowering pH by addition of citric acid, pre-soaking in water, addition of antioxidants, and addition of divalent cations, such as calcium chloride. The addition of asparaginase to reduce asparagine concentration prior to cooking has also been successful in some products (Hendriksen et al., 2009). Nevertheless, the latest report from the European Food Safety Authority (EFSA) on acrylamide levels in foods showed that several hundred μg kg^−1^ (parts per billion) were still present in cereal-based foodstuffs by 2010 (Table 1; European Food Safety Authority, 2012) and processors remain vulnerable to fluctuations in the acrylamide-forming potential of the crop products that make up their raw material.

**Table 1:** Results of EFSA monitoring of acrylamide levels in different cereal-based foods in 2010 (European Food Safety Authority, 2012).

Sample description	*n*	Median (μg kg^−1^)	Mean (μg kg^−1^)	P95 (μg kg^−1^)	Max (μg kg^−1^)
Biscuits, crackers, crisp bread etc.					
Total	462	129	333	1337	5849
Crackers	64	139	178	491	1062
Wafers	37	225	389	1300	1300
Crispbread	54	110	249	1443	1863
Gingerbread	207	134	415	1635	3191
Other	100	99	289	1061	5849
Bread					
Soft Bread	150	18	30	94	425
Breakfast cereals	174	91	138	353	1290
Cereal-based baby food					
Total	128	24	51	175	578
Biscuits and rusks	46	57	86	250	470
Other	82	13	31	130	578
Pastries and cakes	81	55	146	793	890
Muesli and porridge	14	56	80	420	420

The concentrations of acrylamide in food are much lower than those typically used in rodent toxicology studies (Friedman, 2003). Nevertheless, the Food and Agriculture Organisation of the United Nations and the World Health Organisation (FAO/WHO) Joint Expert Committee of Food Additives (JECFA) recently concluded that the margins of exposure (MOE) for acrylamide indicate that its presence in the human diet is a concern and that epidemiological studies are required to estimate the risk (Joint FAO/WHO Expert Committee of Food Additives, 2011) (the MOE is defined by EFSA as the ratio of the level at which a small but measurable effect is observed to the estimated exposure dose; it is also sometimes defined as the ratio of the maximum no-adverse-effect-level to the estimated exposure dose). To date, however, the results of epidemiological studies have been inconsistent. A recent meta-analysis of epidemiological data, for example, led the authors to conclude that there was no relationship between dietary acrylamide intake and cancer (Lipworth et al. 2012); in contrast, a later Danish study did find a link between acrylamide exposure and breast cancer-specific mortality (Olsen et al. 2012). Another recent study showed a link between haemoglobin adducts of acrylamide and glycidamide in umbilical cord blood (reflecting exposure in the last months of pregnancy) and low birth weight and head circumference in babies (Pedersen et al., 2012).

Epidemiology studies can be confounded by extraneous factors, leading to associations being made between the exposure being studied and an effect when the effect actually arises from other differences between the study groups (Hennekens and Buring, 1987). However, the general public are unlikely to be aware of such considerations. The Pedersen et al. (2012) study attracted national media coverage in the United Kingdom, and clearly such findings have the potential to alarm consumers. It would also be complacent for anyone in the food supply chain to assume that regulators will not act in response to such findings. JECFA has already recommended that acrylamide levels in food be reduced as a matter of priority and the European Commission issued ‘indicative’ levels for acrylamide in food in early 2011, then revised them in 2013, with levels for many products being lowered (European Commission, 2011, European Commission, 2013) (Table 2). Indicative values are not safety thresholds, but are intended to indicate the need for an investigation into why the level has been exceeded. The Commission set the levels at what it considered the industry should be able to achieve, based on the results of its acrylamide monitoring programme. However, it is doubtful whether journalists or consumers understand the difference between an indicative value and a safety threshold and the experience in the United Kingdom is that products that are found to contain acrylamide exceeding the indicative value (Table 2) attract negative and potentially damaging publicity.

**Table 2:** Indicative values set by the European Commission for acrylamide content in cereal-based foods in 2011 (European Commission, 2011), proportion of samples exceeding the 2011 indicative values in surveys by the European Food Safety Authority (European Food Safety Authority, 2012) and revised indicative values issued in 2013 (European Commission, 2013).

Food	Indicative value 2011 (μg kg^−1^ (ppb))	Proportion of samples exceeding indicative value (%)		Food sub-category defined in 2013	Indicative value 2013 (μg kg^−1^ (ppb))
		2007–2009	2010		
Soft bread	150	7	3	Wheat-based bread	80
				Other	150

Breakfast cereals (excluding muesli and porridge)	400	6	3	Bran, wholegrain; gun-puffed	400
				Wheat and rye-based	300
				Maize, oat, spelt, barley, rice-based	200

Biscuits, crackers, wafers, crisp bread and similar products	500	8	12	Biscuits, wafers and similar	500
				Crackers	500
				Crisp-bread	450
				Ginger-bread	1000

Biscuits and rusks for infants and young children	250	9	7		200

Other processed cereal-based foods for infants and young children	100	11	6		50

In the USA, the Food and Drug Administration (FDA) has issued an ‘action plan’ on acrylamide with the goals of developing screening methods, identifying means to reduce exposure, assessing dietary exposure of American consumers, increasing understanding of acrylamide toxicology to enable quantitative risk assessment, and informing consumers. To date the FDA has stopped short of issuing advice or restrictions on levels, but in 2005 the Attorney General of the State of California filed a lawsuit against four food companies for not putting a Proposition 65 warning label on their products to make consumers aware that the products contained acrylamide (the State of California requires that a Proposition 65 warning be included in the labelling of any product that contains a compound that may cause cancer, birth defects, or reproductive harm). The lawsuit was settled in 2008 when the companies committed to cutting the level of acrylamide in their products to below 275 μg kg^−1^ and paid $3 million in fines. Note that, as with Europe's indicative levels, there is no evidence that the 275 μg kg^−1^ figure is safe or unsafe; it is simply the figure that the two sides agreed should be achievable, after lengthy negotiation.

It is stating the obvious to say that food producers want to avoid unfavourable headlines, lawsuits and fines, and cereal breeders and growers must take the acrylamide issue seriously because using an alternative raw material, for example by changing to a different variety or even a different crop in some circumstances, may be a relatively easy way for food manufacturers to reduce the level of acrylamide in their products.

Dietary intake of acrylamide for European adults is estimated to be 0.31–1.07 μg per kg of body weight per day, with the intake for adolescents and children being even higher (European Food Safety Authority, 2011a). Cereal foods make a significant contribution to this intake, with dietary preferences between different European countries affecting the total contribution of cereal products and the importance of different food categories within the cereal group. Table 3 shows data on the contribution of different cereal foods to acrylamide intake in Europe's three most populous countries: France, Germany and the United Kingdom. Sweden is also included because it shows some interesting contrasts with the other countries. The data were extracted from a dataset compiled by EFSA (European Food Safety Authority, 2011a).

**Table 3:** Contribution of cereal products (%) to dietary acrylamide intake for adults (18–64) in the UK, France, Germany and Sweden (European Food Safety Authority, 2011a).

Country	Food group					Total
	Biscuits	Crisp bread	Bread^a^	Breakfast cereal	Muesli	
France	7.6	5.3	25.7	1.3	1.0	40.9
Germany	6.1	4.0	32.0	1.2	2.1	45.4
Sweden	5.0	9.7	11.9	1.5	13.1	41.2
United Kingdom	6.3	2.0	15.0	5.0	3.6	31.9

a Includes ‘soft’ and ‘unspecified’ bread types.

The figures in Table 3 shatter two myths: the first that dietary acrylamide is a problem principally for the potato and coffee industries, the second that bread is not a major contributor to dietary acrylamide because acrylamide levels in bread are significantly lower than in some other foods (Table 1). Cereal products account for 31.9–45.4% of dietary acrylamide in the four counties shown, and bread alone for between 11.9 and 32.0%, its relatively low acrylamide content offset by its high consumption levels. The popularity of rye crisp-breads in Sweden is reflected in the data for that country, with crisp-breads contributing 9.7% of total dietary intake, compared with only 2% in the United Kingdom.

Muesli is a significant contributor in all countries but particularly Sweden, where it accounts for 13.1% of total intake. Muesli has many recipes and ingredients but it is traditionally based on uncooked, rolled oats. However, in some cases the oats are lightly toasted and it may be in this process that acrylamide is generated. A similar product, granola, is made by mixing rolled oats with crushed nuts, honey and rice and toasting them in oil, and this would almost certainly produce acrylamide. EFSA also often puts porridge in the same category as muesli, presumably because it too is made from rolled oats. However, porridge is made by boiling rolled oats in water or milk, so it would not contain acrylamide unless the oats were baked or toasted first, which is not the traditional recipe.

### 3.3 Free asparagine concentration is the major determinant of acrylamide-forming potential in wheat and rye

**Fig. 2:**
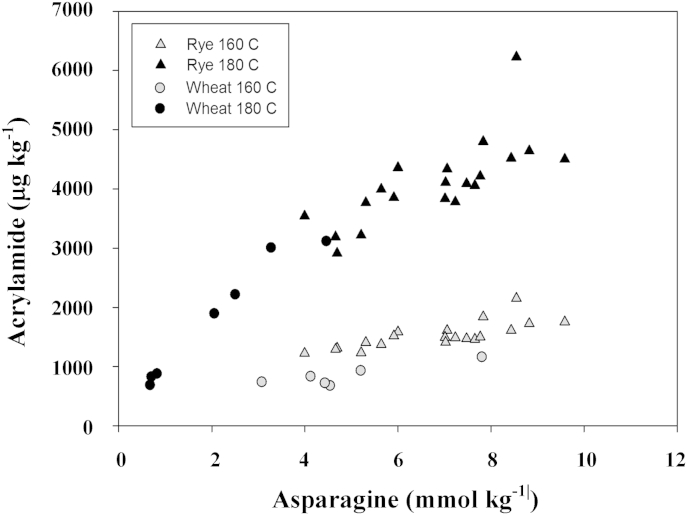
Relationship between free asparagine concentration and acrylamide formation in wheat and rye flour heated for 20 min at 160° or 180 °C (Curtis et al., 2009, Curtis et al., 2010, Muttucumaru et al., 2006, Postles et al., 2013; rye heated at 180 °C data unpublished).

The Maillard reaction is complex, with amino acids participating in the initial reaction with reducing sugars and subsequently with the carbonyl compound intermediates that result from sugar breakdown. The relationship between the concentrations of reducing sugars, sucrose (which can participate if it first undergoes hydrolysis), free asparagine and other free amino acids (which potentially can both drive the first stages of the reaction, resulting in more carbonyl intermediates being produced, and compete with free asparagine for reacting with those intermediates in the later stages of the reaction) may therefore be difficult to predict in some food matrices. Potato is an obvious example, with the relationship between precursor concentration and acrylamide formation even differing between French fry- and crisping-type varieties (Halford et al., 2012a). However, the situation in wheat and rye appears to be more simple, with several studies showing a strong relationship between free asparagine concentration and acrylamide formation (Curtis et al., 2009, Curtis et al., 2010, Granvogl et al., 2007, Martinek et al., 2009, Muttucumaru et al., 2006, Postles et al., 2013) (Fig. 2). It has been suggested that more acrylamide forms per unit of free asparagine in wheat than in rye (Curtis et al., 2010), but the result on which this was based now appears to have been affected by the cooking conditions that were used because a subsequent study did not draw the same conclusion (Postles et al., 2013) and Fig. 2 shows the relationships between free asparagine and acrylamide formation to be very similar in the two flours. Nevertheless, the overall conclusion to be drawn from these studies is that genetic and agronomic approaches to reducing free asparagine accumulation in wheat and rye grain should be pursued to help address the problem of acrylamide formation in foods (Halford et al., 2012b, Muttucumaru et al., 2008). To our knowledge, the results of similar studies on maize, oats or other cereals are not in the public domain. It is likely that food companies have carried out such studies but the knowledge has not been shared. We anticipate that other cereals will be similar to wheat and rye, but this needs to be shown experimentally.

### 3.4 Agronomic and genetic approaches to reducing free asparagine accumulation in cereal grain

There are short-, medium- and long-term approaches to the reduction of free asparagine levels in cereal grain, which should lead to a gradual reduction in acrylamide-forming potential if adopted. These are:

• Identification of varieties with low acrylamide-forming potential
• Identification of genotypes with low acrylamide-forming potential that are not currently cultivated but could be adopted for cultivation or incorporated into breeding programmes
• Identification of environmental factors, including crop management, that affect acrylamide-forming potential
• Development of a comprehensive understanding of asparagine metabolism and its genetic control, including modelling of the pathways and interactions that are involved
• Identification of key quantitative trait loci (QTL), genes and markers that plant breeders could use to produce very low acrylamide varieties

Significant effects of both variety and environment have been demonstrated in wheat and rye. Muttucumaru et al. (2006), for example, analysed the grain of three wheat varieties grown under glass: Solstice, Malacca and Claire. When the plants were fully supplied with nutrients, free asparagine concentration in the grain ranged from 4.12 mmol kg^−1^ in Claire to 4.54 mmol kg^−1^ in Solstice and 5.20 mmol kg^−1^ in Malacca, and acrylamide in flour heated to 160 °C for 20 min ranged from 679 μg kg^−1^ in Solstice to 934 μg kg^−1^ in Malacca, a difference of 38%. This difference shows the potential for varietal selection in addressing the acrylamide problem, but was dwarfed by the effect of sulphur (S) supply, with free asparagine concentration increasing more than 11-fold in Claire, 16-fold in Solstice and almost 30-fold in Malacca as a result of S deficiency, with the acrylamide formed in heated Malacca flour rising to 5198 μg kg^−1^.

**Fig. 3:**
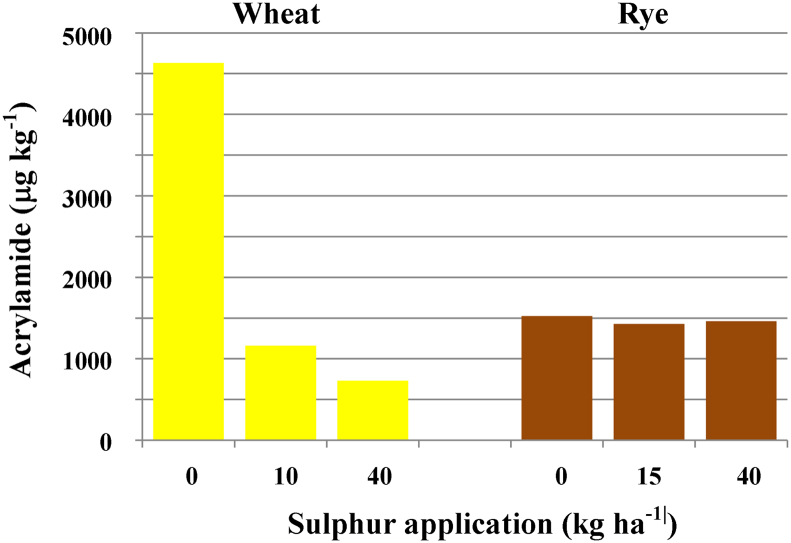
Effect of sulphur availability on the acrylamide-forming potential of wheat and rye grain. The graph shows acrylamide formation in flour prepared from the grain of wheat and rye plants grown with different levels of sulphur application, as indicated, and heated to 160 °C for 20 min. Data from Muttucumaru et al. (2006) and Postles et al. (2013).

The effect of sulphur availability was also shown in field-grown wheat cv. Hereward (Muttucumaru et al., 2006). A field trial was conducted at a site with very poor nutrient retention and grain from two plots treated with 40 kg S ha^−1^ contained 4.43 mmol kg^−1^ and 3.07 mmol kg^−1^ of free asparagine, compared with 7.8 mmol kg^−1^ in grain from plots treated with 10 kg S ha^−1^ and 75.7 and 55.5 mmol kg^−1^ in grain from two plots that did not receive any S fertiliser. Acrylamide formation in heated flour ranged from 723 μg kg^−1^ in a 40 kg S ha^−1^ plot to 5286 μg kg^−1^ in one of the plots that did not receive any S fertiliser, a more than seven-fold difference (Fig. 3).

Granvogl et al. (2007) and Curtis et al. (2009) also showed the dramatic effect of sulphur deficiency on free asparagine concentration and acrylamide-forming potential in wheat. Granvogl et al. (2007) used one cultivar, Star, with acrylamide formation in flour heated at 170 °C for 20 min increasing from a very low 94 μg kg^−1^ with S feeding to 3124 μg kg^−1^ with S deprivation, a 33-fold difference. Curtis et al. (2009) used two varieties, Spark and Rialto, and four doubled haploid lines from a Spark × Rialto mapping population. The study showed not only the effect of the treatment (environment, E), but also the effect of genotype (G) and a strong G × E interaction. For Rialto and three of the doubled haploid lines, for example, the free asparagine concentrations in the S-deprived grain were 12- to 14-fold higher than in the corresponding S-fed grain. For Spark and the other doubled haploid line, on the other hand, the difference was much greater, the free asparagine concentrations in the S-deprived grain being 23- to 24-fold higher than in the corresponding S-fed grain. The grain also accumulated free glutamine in response to sulphur deprivation, although to a lesser extent than free asparagine, and the total free amino acid pool became much larger, possibly due in part to a reduction in storage protein gene expression (Shewry et al., 1983, Shewry et al., 2001).

It should be noted that the levels of acrylamide obtained in these studies reflect the potential of the material to form acrylamide, not how much would be formed in a particular food product. The amount of acrylamide that forms during food production depends not only on the raw material but also on the processing methods and the degree of refining: products made from white flour contain considerably less acrylamide than equivalent products made from wholemeal flour (Claus et al., 2008).

S deficiency had been shown to have a similar effect on barley many years before these studies were published, and long before its significance for food safety was understood (Shewry et al., 1983). At that time and for the next two decades the interest was in the effect of S deficiency on grain protein content and processing properties (Shewry et al., 1983, Shewry et al., 2001, Zhao et al., 1999). The importance of S nutrition for grain quality was clear, but S nutrition of crops was not seen as a problem because, ironically, it had benefited from an atmospheric pollutant, SO_2_, which was generated predominately from the burning of fossil fuels. While being environmentally damaging in other ways, the deposition of S in the form of ‘acid rain’ helped to maintain the S content of soils used for crop production and masked the effect of switching from ‘old-fashioned’ fertilizers such as ammonium sulphate and superphosphate (a mixture of CaSO_4_·H_2_O and Ca(H_2_PO_4_)_2_·H_2_O) to fertilizers based on nitric acid, which are better sources of nitrogen (N) but contain no sulphur. However, in the latter part of the 20th Century there was a huge increase in the use of low sulphur or sulphur-free fuels such as natural gas, and coal- and oil-burning power stations were fitted with pre- and post-combustion systems for removing sulphur. At the same time, higher-yielding crops were denuding soils of minerals, including sulphur, more rapidly (Zhao et al., 1999). As a result, by the end of the 20th Century, increasing areas of land were beginning to become sulphur deficient, affecting the United Kingdom, Northern and Western Europe, Australia and New Zealand (Blair, 2002, Zhao et al., 1999, Zhao et al., 2002).

Even so, Granvogl et al. (2007) argued that S-deficient wheat grain did not pose a high risk for the consumer because it would not be suitable for making food products and would be used for other purposes, such as animal feed. We disagree with this view for two reasons: firstly, the huge increase in free asparagine concentration and acrylamide-forming potential in severely sulphur-deprived wheat is so great that even a small proportion of sulphur-deficient grain in a consignment significantly increases its acrylamide-forming potential. This makes the even distribution of S fertilizer over an entire field very important. Secondly, even moderate S deficiency causes a significant increase in acrylamide-forming potential, with, for example, more than 30% more acrylamide forming in heated flour from wheat treated with 10 kg S ha^−1^ compared with 40 kg S ha^−1^ (Muttucumaru et al., 2006) (Fig. 3). Sulphur deficiency also affects the relative amounts of asparagine in different milling fractions (Shewry et al., 2009): under normal conditions, most asparagine accumulates in the bran fractions but under sulphur deficiency it also accumulates in the white flour fractions and therefore affects more products. The Rothamsted Research recommended level of sulphur for wheat cultivation in the United Kingdom is 20 kg per hectare (Fangjie Zhao, Rothamsted Research, personal communication), which is at the high end of the range recommended by the government's Department for Environment, Food and Rural Affairs (Fertiliser Manual, 2011). The level of 20 kg per hectare is thought to be necessary for high yielding wheat and particularly to ensure the protein quality of bread-making varieties (Zhao et al., 1999). More research is being done to establish optimum S levels to minimise acrylamide-forming potential without imposing unnecessary expense on farmers. In the meantime, farmers are advised to follow this recommendation.

S availability is not the only environment/management factor that affects free asparagine concentration (reviewed by Lea et al., 2007). Nitrogen (N) availability, for example, correlates positively with both the free asparagine and the total free amino acid content, with the effect being greater if the N is applied late in the season (Martinek et al., 2009, Winkler and Schön, 1980). Fertilizing with N will therefore exacerbate the effect of S deficiency, and the deficiency of other minerals, such as potassium, phosphorus and magnesium (Almeida et al., 2000, Nikiforova et al., 2006, Possingham, 1956, Rabe and Lovatt, 1986, Rufty et al., 1990, Stewart and Larher, 1980). This suggests that some plants, including wheat, use free asparagine as an N store (Lea et al., 2007) when deficiencies in other minerals, particularly S, mean that efficient synthesis of storage proteins is not possible (Shewry et al., 2001).

Exposure to toxic metals such as cadmium can also cause accumulation of free asparagine and other amino acids in some plant species (Costa and Morel, 1994: Costa and Spitz, 1997), as can other abiotic stresses such as drought and salt stress (Garthwaite et al., 2005, Good and Zaplachinski, 1994). Free asparagine concentration may also increase in response to pathogen attack and Yellow rust infection has been shown to cause an approximately 50% increase in asparagine synthetase activity in wheat (Kudiyarova et al., 2013).

Environmental effects on free asparagine accumulation were evident in the study by Curtis et al. (2009), which included an analysis of grain from six different varieties grown at six different locations around the UK in 2006 and 2007. Free asparagine concentrations ranged from 0.6 to 4.4 mmol kg^−1^, an almost seven-fold difference, and significant variance arose from location and harvest year, despite similar agronomic practice being followed at each site. The environmental conditions that affected free asparagine concentrations could not be identified: Climate may have been a factor because the summer of 2006 was considerably drier, warmer and sunnier than that of 2007, particularly during the months of June and July, but no trends emerged for soil type.

Despite the contribution of environmental factors (E) to the variance in acrylamide-forming potential described in this study, significant differences were also evident between the varieties, showing the effect of genotype (G), and there were also significant G × E interactions (Curtis et al., 2009). The best-performing variety was Einstein, with an average free asparagine concentration over all locations and both harvest years of 1.89 mmol kg^−1^, compared with 2.59 mmol kg^−1^ for the worst performer, Robigus, a difference of 37%. This again shows the potential of varietal selection in addressing the acrylamide issue. However, it should be noted that Robigus had a low free asparagine concentration in some of the trials, showing the importance of testing varieties with apparently low acrylamide-forming potential under a range of conditions. It is also worth pointing out that food companies would have to assert more influence on the wheat supply chain than they currently do in order to benefit from varietal selection.

G and G × E effects were also evident in the analysis of Spark × Rialto doubled haploid lines that was undertaken in the same study (Curtis et al., 2009). One of the doubled haploid lines contained a significantly lower free asparagine concentration than either parent, which suggests that there is scope for reducing free asparagine concentration in wheat and that breeding could play an important role in mitigating acrylamide risk. It is important that breeders are encouraged to include low free asparagine concentration as a target in their breeding programmes and are provided with the knowledge and tools with which to achieve it.

Providing these tools will require the identification of the factors that control asparagine metabolism and how they interact, and elucidation of the underlying genetics. The enzyme that synthesises asparagine is asparagine synthetase, which catalyses an ATP-dependent transfer of ammonia to aspartate, yielding asparagine (Lea and Fowden, 1975; reviewed by Lea et al., 2007). The reaction requires magnesium ions and the energy-producing hydrolysis of ATP. Asparagine synthesis occurs by amidation of aspartate using either glutamine or ammonium as an amino donor. This means that glutamine synthetase, glutamate dehydrogenase and aspartate aminotransferase are also potentially influential, as is asparaginase, which converts asparagine to aspartate and ammonia (which is recycled), and aspartate kinase, which phosphorylates aspartate in the first step in the biosynthesis of methionine, lysine and threonine.

Asparagine synthetase is difficult to extract in an active form (Joy et al., 1983, Kudiyarova et al., 2013, Snapp and Vance, 1986) because plant tissues contain endogenous natural inhibitors (Rognes, 1980) and relatively high activities of asparaginase and glutamine synthetase, which break asparagine down or compete for substrates (Sieciechowicz et al., 1988). Two asparagine synthetase-encoding genes, *TaASN1* and *TaASN2*, have been identified in wheat (Wang et al., 2005), but may not represent the whole gene family because Arabidopsis, for example, contains three genes, *AtASN1-3*, that are differentially regulated (Lam et al., 1998). Clearly, a comprehensive characterisation of the wheat asparagine synthetase gene family and its regulation would be required before any genetic intervention could be made. Nevertheless, asparagine synthetase is an obvious target because of its central role in asparagine synthesis and the fact that it has already been manipulated successfully to reduce acrylamide-forming potential in GM potatoes (Chawla et al., 2012, Rommens et al., 2008). Genetic intervention to change the activity of asparagine synthetase or other enzymes that affect the relationship between glutamine and asparagine also has the potential to produce varieties that accumulate glutamine instead of asparagine under stress conditions such as sulphur deprivation. The predominant product of the Maillard reaction from glutamine is 2-pyrrolidinone, which is not considered toxic (Stadler et al., 2003). Reducing acrylamide formation by altering the ratio of free asparagine to other amino acids, such as glutamine, rather than reducing the total free amino acid pool, is also attractive because it is likely to have less impact on flavour and colour formation during cooking and processing (Elmore et al., 2008).

As well as directly manipulating asparagine synthetase gene expression, it may be possible to interfere with its regulation, for example in its response to S deprivation. One possible candidate for involvement in the signalling pathway that is responsible is a regulatory protein kinase related to general control non-derepressible-2 (GCN2) of yeast (*Saccharomyces cerevisiae*). In yeast, GCN2 is activated in response to low free amino acid levels and acts to restore amino acid homeostasis by reducing protein production while increasing amino acid biosynthesis. GCN2 homologues have been described in Arabidopsis (Lageix et al., 2008, Zhang et al., 2003, Zhang et al., 2008) and wheat (Byrne et al., 2012) and are present in every plant species for which genome data is available (Halford, 2006). In transgenic wheat plants constitutively over-expressing the wheat homologue, *TaGCN2*, asparagine synthetase gene expression is dramatically reduced and no longer inducible by sulphur deprivation, whereas it is highly responsive to sulphur deprivation in wild-type wheat (Byrne et al., 2012). Asparagine synthetase gene expression in Arabidopsis is also influenced by another regulatory protein kinase, SnRK1 (reviewed by Hey et al., 2010), which controls the response of an asparagine synthetase gene, *ASN1*, to light and sugars (Baena-Gonzalez et al., 2007), possibly through transcription factor bZIP11 (reviewed by Hummel et al., 2009).

An alternative genetic approach would be to identify quantitative trait loci (QTL) that contribute to variance in grain asparagine concentration. The fact that a doubled haploid line from a cross between cultivars Spark × Rialto was shown to have a lower free asparagine concentration in the grain than either parent is encouraging (Curtis et al., 2009) and analysis of the whole population from that cross may enable the identification of useful QTL. Analyses of additional doubled haploid populations from other crosses may then be used to confirm and narrow these QTL and identify additional ones. This approach holds much promise but the identification and testing of robust QTL that could be provided to plant breeders with confidence is still some years away.

The factors that determine acrylamide-forming potential in rye have also been investigated (Curtis et al., 2010, Postles et al., 2013). The results of these studies indicated significant genetic (G) and environmental (E) effects and G × E interactions in the determination of acrylamide precursor levels in rye, as in wheat, but also revealed surprising and important differences between the two closely-related cereals.

The major limiting factor in acrylamide formation in rye flour is free asparagine (Fig. 2), and free asparagine concentration in rye grain is under genetic as well as environmental control, just as in wheat (Curtis et al., 2010, Postles et al., 2013). Curtis et al. (2010) measured free asparagine concentrations in the grain of old and new rye varieties grown for the EU FP6 HEALTHGRAIN diversity programme (Ward et al., 2008). The grain was produced at locations in Hungary, France, Poland and the United Kingdom and harvested in 2005, 2006 and 2007, with both country of origin and year of harvest having significant effects on free asparagine concentration. There was also a statistically significant three-way interaction between country of origin, year of harvest and variety (G × E). The contribution of G to the overall variance in free asparagine concentration suggested that the trait may be amenable to improvement by breeding.

The study showed that total free asparagine in the grain was related to bran yield, the concentration being higher in grain giving a higher proportion of bran on milling. It also enabled old and new varieties of rye to be compared, with varieties currently being used for commercial production generally having lower levels of free amino acids, particularly asparagine and proline, than older varieties. It is not clear why this should be because free amino acid concentration has not been a target for selection in rye breeding programmes up to now.

This study was not designed to assess the effect of different fertilization regimes on acrylamide-forming potential. However, this was the objective in a subsequent analysis of five current commercial varieties of rye grown in the same location in the United Kingdom at the same time, with nine different combinations of S and N application (Postles et al., 2013). High levels of N application (200 kg ha^−1^) significantly increased free asparagine and total free amino acid concentration and acrylamide formation during heating. S application mitigated the effect of high N application in two of the varieties, but there was no direct effect of S, in stark contrast with wheat (Fig. 3). Rye may be better able to scavenge available S from the soil than wheat (Bushuk, 2001), but may also respond differently to S deficiency: it appeared to accumulate less N in the grain when it had insufficient S available, rather than use free asparagine as an alternative N store (Postles et al., 2013).

### 3.5 Summary on strategies to keep acrylamide-forming potential as low as reasonably achievable in wheat and rye

Work continues on improving our understanding of the factors that affect acrylamide-forming potential in wheat and rye grain, but some advice can already be given to farmers, breeders and food processors with some confidence:

• There are significant differences between varieties (genotypes) of both wheat and rye with respect to acrylamide-forming potential. Selecting low acrylamide varieties for the production of foods in which acrylamide is likely to form would be a beneficial and relatively straight-forward step in reducing acrylamide levels in cereal-based foods.
• The limiting factor for acrylamide-forming potential in wheat and rye is free asparagine concentration. This is the parameter on which varietal selection should be based and a trait that should be incorporated into breeding programmes if it is not already.
• Environmental factors (E) have significant effects on free asparagine accumulation in both wheat and rye, on their own and in combination with varietal differences (G × E). It is important that varieties are tested in a range of conditions before being categorised as low in acrylamide-forming potential.
• Sulphur (S) deficiency causes a massive accumulation of free asparagine in wheat grain and farmers should be encouraged to ensure that sulphur deficiency is avoided. The Rothamsted Research recommendation for S application is 20 kg S ha^−1^ and this should be followed while more research is being undertaken to establish the optimum level for keeping acrylamide-forming potential as low as reasonably achievable. The even distribution of S fertilizer over a field right up to the margins is important.
• S deficiency does not appear to affect free asparagine concentration and acrylamide-formation potential in rye, at least under field conditions, so decisions on S fertilization of rye can be made without consideration of the acrylamide issue.
• Nitrogen (N) fertilization increases free asparagine and total free amino acid concentration in both wheat and rye, causing a concomitant increase in acrylamide-forming potential. N fertilizer is required to maintain the yield and quality of the crop, but excessive application should be avoided. Ensuring that other minerals are available to the crop may mitigate the effect of excessive N.

Some or all of these points may apply to other cereals. Barley, for example, certainly accumulates free asparagine in response to S deficiency just as wheat does (Shewry et al., 1983). However, relatively little work has been done on maize or rice. This is perhaps particularly surprising for maize given the popularity of tortillas, fried tortilla chips and other maize-based snacks that contain acrylamide. Harrigan et al. (2007) did measure the concentration of free asparagine and other metabolites in different maize varieties grown at different locations but did not discuss the implications of the results for acrylamide formation in fried and baked products.

## 4 Furan and related contaminants

Furan is a heterocyclic compound consisting of a five-membered aromatic ring comprising four carbon atoms and one oxygen (Fig. 1), and more complex compounds containing such rings are also referred to as furans. Furan causes liver cancer in rodents (Leopardi et al., 2010) and is classed by the International Agency for Research on Cancer as ‘possibly carcinogenic’ to humans (Group 2B) (International Agency for Research on Cancer, 1995). As with acrylamide, the actual risk posed by the presence of very low levels of furan in food is not known, but the low margin of exposure indicates a cause for concern and makes it sensible for dietary intake to be reduced if possible (Bakhiya and Appel, 2010, Moro et al., 2012).

Furan forms through several pathways (Crews and Castle, 2007, Moro et al., 2012, Perez Locas and Yaylayan, 2004), one of which is the thermal degradation of polyunsaturated fatty acids (PUFAs). As described in Section 2, PUFAs are prone to oxidation during cooking and high-temperature processing, giving rise to lipid peroxides. One of these, 4-hydroxy-2-butenal, can form furan through cyclization and dehydration (Moro et al., 2012). Another route is within the Maillard reaction, whereby the thermal degradation of free amino acids in the presence of reducing sugars gives rise to glycolaldehyde and acetaldehyde. These intermediates undergo aldol addition to 2-deoxyaldotetrose, which further reacts to form furan. Furans can also form directly from sugar breakdown, via the intermediates, 1-deoxyosone and 3-deoxyosone, and aldotetrose and its derivatives, 2-deoxyaldotetrose, and 2-deoxy-3-ketoaldotetrose. Degradation of ascorbic acid and dehydroascorbic acid also gives rise to furan via aldotetrose and its derivatives.

There has been most concern over the levels of furan in coffee, with a recent EFSA survey finding particularly high levels (mean 158 μg kg^−1^) in Espresso-type coffees (European Food Safety Authority, 2011b). In the same survey, the mean furan content of cereal-based baby foods and other cereal products was 23–25 μg kg^−1^ and 15–18 μg kg^−1^, respectively. Cereal-based baby foods contribute 3% of the dietary furan intake in infants and 12% in toddlers, while other cereal products contribute 7.7% of the dietary intake of older children and 10% of the intake of adolescents (European Food Safety Authority, 2011b). In adults, dietary intake from cereal products becomes less important because it is dwarfed by that from coffee.

As with acrylamide, furan formation can be reduced by modifying food processing conditions, such as temperature, time, phosphate concentration and pH (Fan et al., 2008). Ferric ions have been shown to increase its formation from linoleic acid, while addition of reducing agents decreases its formation from ascorbic acid (Mark et al., 2006). As far as we are aware, no attempt has been made to compare the furan-forming potential of different crop varieties, or to devise strategies for reducing it through breeding. Given that one route for its formation is through the Maillard reaction it is likely that low reducing sugar and free amino acid concentrations in the raw material would be desirable. Efforts to improve the heat stability of plant oils by reducing the proportion of unsaturated fatty acids may also reduce the potential for furan formation. However, the other main precursor is ascorbic acid, which is, of course, Vitamin C, an essential part of the human diet.

## 5 Hydroxymethylfurfuryl

One of the compounds containing a furan ring that has attracted some attention in cereal products is hydroxymethylfurfuryl (HMF) (Ramírez-Jiménez et al., 2000), which consists of a furan ring with both aldehyde and alcohol functional groups (Fig. 1). HMF arises via the dehydration of fructose (Román-Leshkov et al., 2006). It is a common contaminant of dark beers (Husøy et al., 2008) and is sometimes used as an indicator of excessive heat treatment in biscuit manufacture, partly because products containing high levels of HMF may also contain a lot of acrylamide. In humans, HMF is metabolised to 5-hydroxymethyl-2-furoic acid (HMFA), which is excreted in urine, and 5-sulfoxymethylfurfural (SMF). SMF can form adducts with DNA or proteins and rodent toxicology studies have indicated potential toxicity and carcinogenicity (Husøy et al., 2008).

## 6 Conclusions

Foods, and in particular cooked and processed foods, are complex mixtures of compounds and it is a fact of life that some of those compounds may have the potential to be harmful to health. The technology for identifying the individual constituents of foods has developed rapidly over the last decade or so, as has its accessibility to mainstream laboratories, and this trend looks set to continue for the foreseeable future. Foods are therefore coming under closer and closer scrutiny, and it is almost inevitable that more compounds will be identified that are undesirable but present in only tiny quantities. Assessing the risk that tiny amounts of such compounds in the diet represent is unlikely to be any easier than it has been for acrylamide and furan. It is also a difficult issue for the food industry in terms of public perception. These contaminants must have been present in our diet since humans first started cooking food to make it more palatable, and the pressure to take action over them has therefore arisen from new knowledge, not new risk. Nevertheless, many consumers expect their food to be absolutely risk-free, however unrealistic that might be, and are generally unimpressed by risk/benefit arguments. At the same time, they also demand foods with the colours, flavours and aromas that form through similar pathways to acrylamide and furan.

In the case of cereals, the problem is made even more difficult by the fact that any possible risk must be balanced against the established nutritional and health benefits of eating cereal- and particularly wholegrain cereal-derived foods, and the important contribution made to our diet by the products that are affected. Cereal products are a valuable source of energy in the form of complex carbohydrate. Wholegrain wheat products are a rich source of fibre, protein, B vitamins, iron, calcium, phosphoric acid, zinc, potassium and magnesium (Shewry et al., 2009), while rye is also rich in fibre, notably arabinoxylan and β-glucan, as well as beneficial phytochemicals such as folate, phenolic acids, alkylresorcinols (phenolic lipids) and sterols (Nyström et al., 2008). It is important that the benefits of these foods are retained and that consumers are not put off eating wholegrain cereals.

Despite these considerations, regulatory bodies around the world have been gradually increasing the pressure on the food industry to reduce the levels of processing contaminants in foods. As discussed in Section 2, for example, the US government introduced legislation requiring information on *trans* fat content of food products to be included in the Nutrition Facts panel of food labels in 2008, while the European Commission issued indicative levels for acrylamide content in food in 2011 and lowered them for many products in 2013 (Section 3). As a result, the problem of processing contaminants is now one of the most difficult facing the food industry, and cereal breeders and producers need to be aware of this and take action. Indeed, it is our view that those that are failing to do so are out of touch with some of their major end-users.
